# Secretory signal peptide modification for optimized antibody-fragment expression-secretion in *Leishmania tarentolae*

**DOI:** 10.1186/1475-2859-11-97

**Published:** 2012-07-25

**Authors:** Stephan Klatt, Zoltán Konthur

**Affiliations:** 1 Max Planck Institute for Molecular Genetics, Ihnestraße 63-73, 14195, Berlin, Germany; 2 Freie Universität Berlin, Faculty of Biology, Chemistry and Pharmacy, Takustraße 3, 14195, Berlin, Germany

**Keywords:** Secretory signal peptide (SP), SignalP, single-chain Fragment variable (scFv), *Leishmania tarentolae*

## Abstract

**Background:**

Secretory signal peptides (SPs) are well-known sequence motifs targeting proteins for translocation across the endoplasmic reticulum membrane. After passing through the secretory pathway, most proteins are secreted to the environment. Here, we describe the modification of an expression vector containing the SP from secreted acid phosphatase 1 (SAP1) of *Leishmania mexicana* for optimized protein expression-secretion in the eukaryotic parasite *Leishmania tarentolae* with regard to recombinant antibody fragments*.* For experimental design the online tool SignalP was used, which predicts the presence and location of SPs and their cleavage sites in polypeptides. To evaluate the signal peptide cleavage site as well as changes of expression, SPs were N-terminally linked to single-chain Fragment variables (scFv’s). The ability of *L. tarentolae* to express complex eukaryotic proteins with highly diverse post-translational modifications and its easy bacteria-like handling, makes the parasite a promising expression system for secretory proteins.

**Results:**

We generated four vectors with different SP-sequence modifications based on in-silico analyses with SignalP in respect to cleavage probability and location, named pLTEX-2 to pLTEX-5. To evaluate their functionality, we cloned four individual scFv-fragments into the vectors and transfected all 16 constructs into *L. tarentolae*. Independently from the expressed scFv, pLTEX-5 derived constructs showed the highest expression rate, followed by pLTEX-4 and pLTEX-2, whereas only low amounts of protein could be obtained from pLTEX-3 clones, indicating dysfunction of the SP. Next, we analysed the SP cleavage sites by Edman degradation. For pLTEX-2, -4, and -5 derived scFv’s, the results corresponded to *in-silico* predictions, whereas pLTEX-3 derived scFv’s contained one additional amino-acid (AA).

**Conclusions:**

The obtained results demonstrate the importance of SP-sequence optimization for efficient expression-secretion of scFv’s. We could successfully demonstrate that minor modifications in the AA-sequence in the c-region of the natural SP from SAP1, based on *in-silico* predictions following the (-3, -1) rule, resulted in different expression-secretion rates of the protein of interest. The yield of scFv production could be improved close to one order of magnitude. Therefore, SP-sequence optimization is a viable option to increase the overall yield of recombinant protein production.

## Background

*Leishmania tarentolae* is a eukaryotic flagellated unicellular parasite with a broad range of applications [1-3]. An increasing field of interest is its use as a host for recombinant protein expression [4-8]. It allows complex eukaryotic protein expression at high levels compared to other eukaryotes, and has the ability to post-translationally modify proteins. Furthermore, its easy bacteria-like handling makes the parasite a promising expression system for eukaryotic proteins. All these characteristics suggest Leishmania to be a good choice for the expression-secretion of recombinant antibody fragments.

A single-chain Fragment variable (scFv) is the smallest functional entity of a monoclonal antibody consisting of a single-polypeptide. It is composed of the variable regions of the heavy (V_H_) and the light (V_L_) chain of immunoglobulins, which are connected with a flexible amino-acid (AA) linker of varying length [9].

Antibodies, like many other proteins, are naturally secreted. For targeted protein transport, special sequence motifs are usually necessary [10-12]. Secretory signal peptides (SP) function as sorting signals. In general, they are located at the N-terminus of proteins and their length ranges between 15–30 AAs [13]. During translocation across the endoplasmic reticulum membrane, the SP is usually cleaved off and the protein is entering the secretory pathway [11,13,14]. Changes of 2–4 AAs of the SP-sequence can result in new cleavage sites and in changed expression-secretion efficiency in e.g. lactic acid bacteria [15]. Secretory leader sequence optimization has been widely applied in other organisms as well, such as *E. coli*[16], different yeast strains [17,18], or in insect cells using the baculoviral expression system [19].

In this study, we explored the use of *L. tarentolae* as host for protein expression-secretion of four human recombinant scFv’s derived from a semi-synthetic single-framework phage display antibody library [20,21]. To accommodate scFv’s with efficient cleavage sites, we followed a two step strategy. First, we set out to *in-silico* model SP-sequences in combination with suitable restriction sites for cloning using SignalP [22]. Second, we designed appropriate vector constructs to evaluate resulting SP-sequences *in-vivo* for optimized scFv expression-secretion in *L. tarentolae*. As a starting point we utilised the commercial *L. tarentolae* protein expression-secretion vector pLEXSY-sat2 (Jena Bioscience), containing the SP-sequence of secreted acid phosphatase 1 (SAP1 [UniProt:Q25332]) of *L. mexicana*[23]. This SP-sequence has been successfully applied in a similar or the same vector for the expression-secretion of e.g human erythropoietin [4], C-reactive protein [24] and heterotrimeric laminins [25].

## Results and discussion

### *In-silico* analysis of natural secretory signal peptides

A number of online tools are available for predicting signal peptides and corresponding cleavage sites in protein constructs based on their AA sequence [13]. In two comparative studies, the online program SignalP was identified to be the method of choice [26,27]. Thus, we applied this online tool for cleavage site prediction using an algorithm based on Hidden Markov Models (HMM) on the SP-sequence in vector pLEXSY-sat2 in combination with human scFv sequences.

**Figure 1:**
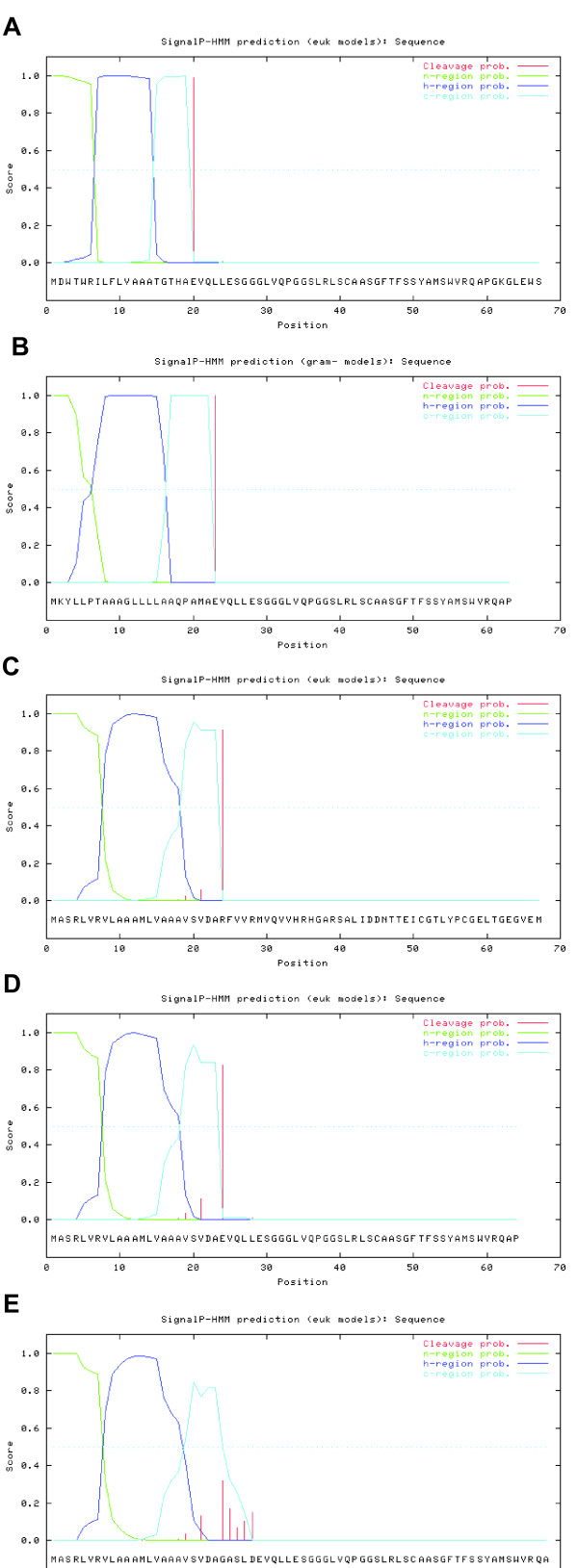
**SignalP 3.0 analysis of different SP-sequences.** Graphical output of SignalP-HMM, showing the posterior probabilities for n-, h- and c-region and cleavage sites. (**A**) human IgG VH leader peptide with scFv for expression in human cells (**B**) *Erwinia carotovora* pelB SP with human scFv for *E. coli* expression; (**C**) *Leishmania mexicana* secreted acid phosphatase 1 (SAP1); (**D**) the SAP1 SP-sequence with human scFv neglecting any cloning strategy; (**E**). the SAP1 SP-sequence in pLEXSY-sat2 with scFv cloned via available XbaI RE-site.

First, we analysed the cleavage site for natural human IgG expression-secretion with its natural IgG V_H_ leader peptide [UniProt:Q9Y298] [28] in plasma cells using SignalP 3.0 [29] (**MDWTWRILFLVAAATGTHA**_scFv), which resulted in a 100% cleavage prediction at the first AA of the scFv (Figure 1A). In parallel, we compared the cleavage site of scFv constructs with the *Erwinia carotovora* pectate lyase 2 (pelB) leader peptide [UniProt:P0C1C1] [30] (**MKYLLPTAAAGLLLLAAQPAMA**_scFv; Figure 1B). The SP of pelB is frequently used for scFv expression-secretion in *Escherichia coli* and results in the same cleavage site as during natural human IgG expression-secretion. Next, we analysed whether a simple transfer of a scFv into *L. tarentolae* expression-secretion vector pLEXSY-sat2 would yield the same results. Endogenously, the SP of SAP1 (**MASRLVRVLAAAMLVAAAVSVDA**_SAP1) is cleaved off completely. *In-silico*, SignalP 3.0 assigns a probability of ~95% for this cleavage event (Figure 1C). If scFv’s were theoretically to be cloned directly in fusion with the SAP1 SP-sequence neglecting the necessity for a cloning site, the predicted cleavage probability is reduced to ~83% (Figure 1D). In pLEXSY-sat2, the scFv could be cloned downstream of the SP-sequence into the multiple cloning site II (MCS II, underlined in the following) by using the available restriction site XbaI. This would result in the AA-sequence **MASRLVRVLAAAMLVAAAVSVDAGASLD**_scFv. The *in-silico* cleavage prediction of this construct worsens. No unique cleavage site was assigned, rather multiple sites were predicted with similar probabilities ranging only around 10-30% (Figure 1E). Further, none of the cleavage sites were predicted to yield the same cleavage product as *E. coli* or human cells, i.e. targeting the first AA of the scFv (glutamic acid (E); **E**VQLLES…), which is defined as position 1. This is attributed to the fact that the AA composition upstream position 1 (−1 to −3) has an influence on the cleavage site [31]. Additionally, at least one amino-acid is not cleaved off extending the scFv-sequence N-terminally, which could potentially influence protein functionality. For all these reasons we conclude that the use of the pLEXSY-sat2 vector in its current state is not sensible for expression-secretion of scFv’s in *L. tarentolae.* Hence, the vector needs specific modification in regard to cleavage site as well as cleavage probability.

### *In-silico* modification of secretory signal peptide SAP1 for scFv expression

Modification of the SP-sequence to enhance cleavage site probability at the first AA of scFv’s was carried out *in-silico* following two rationales: First, to optimize AA composition defined by the multi cloning site (MCS II) accommodating unique restriction enzyme (RE) sites for scFv cloning. Second, to find a site with the highest possible cleavage probability and lowest number of alternatives at the first AA residue of the scFv. Ideally, fewer cleavage sites predicted from SignalP and a higher probability of only one successful cleavage should lead to better expression-secretion results of the protein.

**Figure 2:**
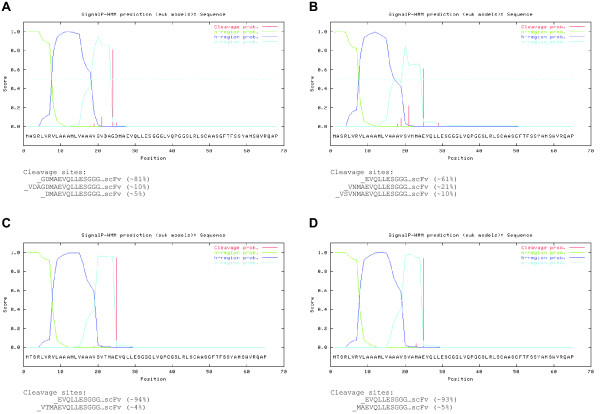
**SignalP 3.0 analysis of novel pLTEX vectors with a scFv.** Graphical output of SignalP-HMM, showing the posterior probabilities for n-, h- and c-region and cleavage sites of pLTEX vectors with scFv for (**A**) pLTEX-2 (**B**) pLTEX-3, (**C**) pLTEX-4 and (**D**) pLTEX-5. Alternative cleavage sites and respective probabilities are displayed.

The scFv’s in our experiments were derived from the phage display vector pIT2, which contains a unique NcoI RE-site upstream of position 1. To enable simple cloning, the MCS II should contain a NcoI RE-site or, alternatively, a RE-site with compatible overhang, such as PciI. Hence, in an iterative process we first set out to modify only the MCSII by introducing changes in the vector pLEXSY-sat2 on the nucleic acid level to accommodate compatible RE-sites for cloning and analysed the results *in-silico* applying SignalP. This approach resulted in the design of pLTEX-2, described in detail below. In a second step, we included the modification of the last AA positions of the SAP1 SP-sequence and conducted the same analyses to obtain the desired characteristics of SP-sequence cleavage at position 1. This approach led to the design of vectors pLTEX-3 to pLTEX-5 (Figure 2). The detailed rational for all four vectors is described in detail below.

pLTEX-2 was designed on the merit to maintain the original SP-sequence of SAP1, only exchanging the AA encoded by MCS II to accommodate a PciI restriction site. The SP-sequence will be cleaved at the identical position as in SAP1 and, hence, the scFv is extended N-terminally by four AAs. pLTEX-2 has a predicted major cleavage site with a probability of ~81% at the glycine (G) residue in position −4 relative to the scFv’s start and two minor cleavage sites at positions −7 and −3 with probabilities of ~10% or below (Figure 2A).

The constructs pLTEX-3 to pLTEX-5 were designed to contain a SP-sequence with a high cleavage probability directly at position 1. To achieve this, the natural SAP1 SP-sequence was shortened by the two AA residues aspartic acid (D) and alanine (A), corresponding to positions −6 and −5 in pLTEX-2. At the same time we introduced variations in the AA residue at position −3 by incorporating asparagine (N), threonine (T) and alanine (A), respectively.

pLTEX-3 contains an asparagine residue at position -3, a predicted major cleavage site with a probability of ~61% at position 1 as well as two additional cleavage sites upstream with lower probabilities (~21% and ~10%; Figure 2B).

pLTEX-4 and pLTEX-5 contain a threonine or alanine residue at position -3 and their predicted major cleavage sites with a probability of close to 95% is at position 1 directly. Both constructs have additional predicted minor cleavage sites with probabilities of below ~5% (Figure 2C,D). The additional modification in pLTEX-4 and pLTEX-5 at the second N-terminal position of the SP-sequence (A to T) was introduced to abolish a NcoI RE-site in the vector and is not considered essential for the functionality of the SP [32].

### Generation of expression-secretion vectors pLTEX-1 to pLTEX-5

**Figure 3:**
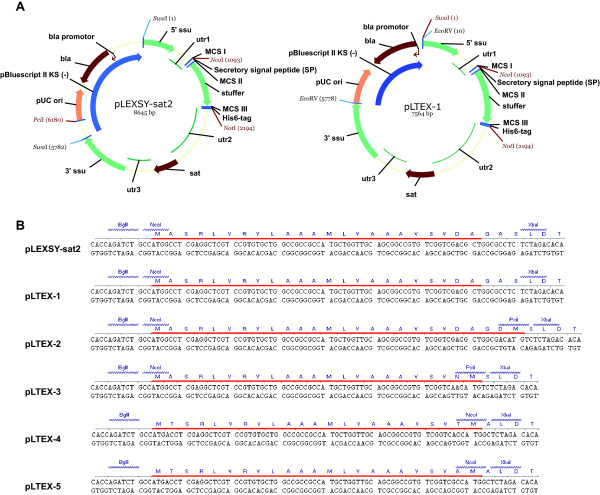
**Description of vectors pLEXSY-sat2 and pLTEX-1 to pLTEX-5.** (**A**) Full vector maps of pLEXSY-sat2 and pLTEX-1. pLEXSY-sat2 (Jena Bioscience) was used to generate pLTEX-1 with a reduced pBluescript II KS (−) backbone and different RE-sites for linearization (SwaI to EcoRV). (**B**) Differences in the SP-sequences and MCS II of vectors pLEXSY-sat2 and pLTEX-1 to pLTEX-5. Protein sequence of SP’s are underlined with red.

Novel vectors were designed based on the above considerations for AA composition of SP-sequences. First, the general transfection strategy in *L. tarentolae* was assessed. To obtain stable expression of recombinant protein a linearised construct needs to be transfected to allow chromosomal integration into a predefined locus by homologous recombination [4]. The original pLEXSY-sat2 vector (Jena Bioscience) is linearised by RE SwaI (Figure 3A). However, all scFv’s used in this study contain a conserved SwaI RE-site. Therefore we replaced SwaI with EcoRV. At the same time we reduced the pBluescript II KS (−) backbone of the vector by 1.1 kb to enhance transformation efficiency in *E. coli* generating vector pLTEX-1 (Figure 3A). Note, that pLTEX-1 in its current state is not suitable for scFv expression according to our *in-silico* analysis alike pLEXSY-sat2, since multiple cleavage sites with poor cleavage probabilities are anticipated. Next, the cloning strategy for scFv was determined. MCS I and MCS II were analysed for the availability of compatible restriction sites and modified to allow cloning of scFv’s via NcoI and NotI. During the same process, modified SP-sequences were introduced using specific primer-sets creating expression-secretion vectors pLTEX-2 to pLTEX-5 (Figure 3B).

In pLTEX-2 and pLTEX-3, a PciI RE-site was introduced into MCS II, since PciI produces a compatible overhang to NcoI. In vectors pLTEX-4 and pLTEX-5, a NcoI RE-site was introduced in MCS II and at the same time another abolished in MCS I.

### Analysis of vector-dependent scFv expression rates *in vivo*

To test the expression-secretion efficiency of the new vectors pLTEX-2 to pLTEX-5, four independent scFv’s were cloned into each vector and transfected into *L. tarentolae*. Stable integration of the expression cassette into the host chromosome was analysed by genomic PCR and could be verified for all 16 constructs (data not shown).

In all pLTEX vectors, protein expression occurs constitutively and was tested for up to four clones per construct. For each construct, the clones showed similar expression levels according to SDS-PAGE analysis and no difference in expression-secretion between single-clones and multi-clones (collection of unknown number of single-clones) from the same vector was observed (data not shown).

**Figure 4:**
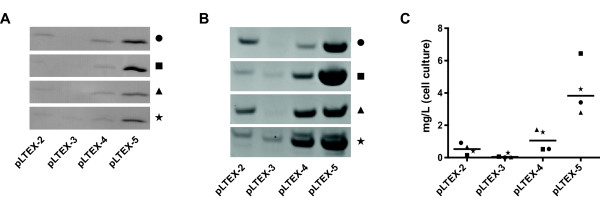
**Expression-secretion analysis of 16 scFv’s in vectors pLTEX-2 to pLTEX-5.** (**A**) Western blot analysis of scFv’s in culture supernatant. Detection of scFv’s with Protein L-HRP (1:5000). (**B**) SDS-PAGE analysis of Ni-NTA purified scFv’s from 100 ml cultures. Proteins are separated on 4-12% Bis-Tris gels (Invitrogen). (**C**) Protein yield from 100 ml culture volumes grown for 72 h of IMAC-purified scFv’s determined in duplicate by densitometrical analysis on SDS-PAGE. Black circles, squares, triangles and stars represent scFv-1 to −4, respectively.

First, we analysed the culture supernatant of all 16 different scFv constructs by Western blot for the presence of scFv and observed concentration differences (Figure 4A). Independent of which scFv was cloned, most protein could be detected in supernatants of clones expressed in pLTEX-5 followed by pLTEX-4, pLTEX-2 and pLTEX-3. The nature of SP-sequence and its corresponding cleavage site can influence the expression-secretion efficiency [15] and has a direct influence on the expression level of scFv’s.

Next, we purified scFv from the culture supernatant of all 16 clones by immobilised metal-ion chromatography (IMAC) and analysed the data by Coomassie-dyed SDS-PAGE (Figure 4B). As expected, the same order of expression efficiency was observed and the intensity of the protein band corresponding to the scFv (~27 kDa) was quantified densitometrically (Figure 4C). The highest expression rate for scFv’s was recorded from clones in vector pLTEX-5, with a median expression of 3.83 mg/L. Clones from vectors pLTEX-4 and pLTEX-2 had a median expression rate of 1.05 mg/L and 0.53 mg/L, respectively. The lowest median expression rate was observed in clones from vector pLTEX-3 (0.04 mg/L).

The observed expression-secretion efficiencies are in good agreement with our knowledge on SP-sequences, formulated as the signal peptidase recognition site or (−3, –1) rule [33] based on the analysis of 78 different natural eukaryotic signal sequences [31]. The preference of a small neutral AA in position −1 and exclusion of a certain large polar, aromatic and charged AA in position −3 can be explained by the nature of the signal peptidase I, which is mainly responsible for SP cleavage. The following ranking was deduced according a ‘best fit’ analysis for AA and signal peptidase I combination for position −1: A > G,S > C,T > Q; for position −3: A > V > C,S,T > L,I > G [33].

In concordance with this, all our vectors follow this general rule. In detail, our choice for alanine in position −1 respectively to the cleavage site in all pLTEX vectors is optimal. However, the AAs in position −3 are different. Here, pLTEX-5 and pLTEX-4 have the preferred small neutral AA (A, T) whereas pLTEX-2 has a hydrophobic AA (V). The SP-sequence of pLTEX-3 has a large polar AA (N) in position −3 resulting in poor expression, possibly due to steric hindrance within the active site of signal peptidase I resulting in low cleavage efficiency. The expression level is in good agreement with the order of the AA observed in position −3 with alanine giving the best expression-secretion rate.

Finally, we analysed the AA-composition of the N-terminus of all 16 obtained scFv’s by Edman degradation [34]. In pLTEX-2 the SP is cleaved off at the predicted glycine residue four AA downstream of the first AA (E) of the scFv. For vectors pLTEX-4 and pLTEX-5 the N-terminus also follows the prediction. The SP is cleaved off at the predicted site, which is glutamic acid (E). Additionally, a second cleavage site could be detected at position −2, extending the scFv’s by two AAs; methionine and alanine (MA). Notably, the AA-composition in position −5 and position −3 also fulfils the signal peptidase recognition site rule, but with low efficiency. The observed frequency of this cleavage isotype has become visible after digesting large quantities of the protein (data not shown).

Surprisingly, the expected *in-silico* cleavage site at the first AA of the scFv could not be confirmed in the samples derived from vector pLTEX-3. The SP was cleaved one AA downstream at an alanine residue extending the scFv’s. We propose that the offset of signal peptidase I occurs as a consequence of the unfavourable AA composition downstream of position 1, resulting in inefficient cleavage explaining the low expression-secretion yield.

## Conclusions

To our knowledge, this study represents the first in detail analysis of a signal peptidase recognition site in *L. tarentolae*. We could successfully demonstrate that minor modifications in the AA-sequence of the natural SP from SAP1 based on *in-silico* predictions, following the (-3, -1) rule resulted in different expression-secretion rates of the protein of interest. Using these general principals the yield of scFv production could be improved close to one order of magnitude.

## Methods

### Generation of expression vectors pLTEX-1 to pLTEX-5

All plasmids and primers used in this study are listed in Tables 1 and 2, respectively. Vector pLTEX-1 (Figure 3A) was generated in three steps: First, the pBluescript II KS (−) backbone (ori for *E. coli*) from pLEXSY-sat2 (Jena Bioscience) was reduced from 2.86 kb to 1.82 kb by PCR amplification using primer 1 and 2. The primers added EcoRV restriction sites to both ends of amplicon 1. Secondly, the chromosomal integration cassette was PCR amplified using primers 3 and 4 (5783 bp) from pLEXSY-sat2. Again, the primers added EcoRV restriction site to both ends creating amplicon 2. Next, amplicon 1 and 2 were digested with EcoRV, ligated, transformed into electrocompetent DH10B cells (Invitrogen) and plated on Ampicillin-containing agar-plates for clonal selection. The obtained vector was named pLTEX-1P (P for precursor). In a final step, the expression cassette was exchanged in pLTEX-1P with a parental fragment from vector pLEXSY-sat2. For this, both vectors were digested with BsrGI and BamHI, respective fragments were gel-purified, ligated and transformed into DH10B cells to generate the expression vector pLTEX-1. Vectors pLTEX-2 to −5 are based on pLTEX-1 and only differ in the SP-sequence. Vector pLTEX-2 was generated from pLTEX-1 and amplicon 3, which was a PCR fragment from pLEXSY-sat2 using primers 3 and 5. Both, PCR fragment and pLTEX-1 were digested with BamHI and XbaI, ligated together and transformed into DH10B cells to generate pLTEX-2. Vectors pLTEX-1 and pLTEX-2 only differ in MCS II that now contains a RE-site for PciI. Vector pLTEX-3 was generated as described for pLTEX-2, except using primer 6 instead of 5. For the generation of pLTEX-4, another precursor (pLTEX-4P) was necessary. First, a PCR amplicon using primers 7 (adding cutting site BglII) and 8 was generated on pLEXSY-sat2 as template. The PCR-fragment and pLTEX-1 were then digested with BglII and KpnI, ligated together and transformed into DH10B to generate pLTEX-4P. In a second step, a PCR amplicon using primers 3 and 9 (adding cutting site NcoI) was generated on pLTEX-4P. This PCR-fragment and pLTEX-1 were digested with BamHI and XbaI, ligated and transformed into DH10B cells to generate pLTEX-4. Vector pLTEX-5 was constructed starting from pLTEX-4P by generating a PCR amplicon using primers 3 and 10 (adding cutting site NcoI). Both, PCR-fragment and pLTEX-1 were digested with BamHI and XbaI, ligated and transformed into DH10B cells to generate pLTEX-5. All new vectors were sequence-verified by DNA sequencing applying the Sanger method.

**Table 1:** Vectors and constructs used in this study

**Name**	**Characteristics**	**Reference**
pLEXSY-sat2	Expression vector pLEXSY-sat2 with sat gene (streptothricine acetyltransferase) allowing selection of recombinant LEXSY strains with antibiotic NTC; and Amp for DH10B	Jena Bioscience (Cat.-No. EGE-234)
pLTEX-1	Based on pLEXSY-sat2; with reduced pBlueScript backbone and changed RE-site for vector linearization prior transfection (SwaI to EcoRV)	this study
pLTEX-2	based on pLTEX-1; changed SP sequence	this study
pLTEX-3	based on pLTEX-1; changed SP sequence	this study
pLTEX-4	based on pLTEX-1; changed SP sequence	this study
pLTEX-5	based on pLTEX-1; changed SP sequence	this study
pLTEX-2_scFv-1	Vector for expression-secretion of scFv-1	this study
pLTEX-3_scFv-1	Vector for expression-secretion of scFv-1	this study
pLTEX-4_scFv-1	Vector for expression-secretion of scFv-1	this study
pLTEX-5_scFv-1	Vector for expression-secretion of scFv-1	this study
pLTEX-2_scFv-2	Vector for expression-secretion of scFv-2	this study
pLTEX-3_scFv-2	Vector for expression-secretion of scFv-2	this study
pLTEX-4_scFv-2	Vector for expression-secretion of scFv-2	this study
pLTEX-5_scFv-2	Vector for expression-secretion of scFv-2	this study
pLTEX-2_scFv-3	Vector for expression-secretion of scFv-3	this study
pLTEX-3_scFv-3	Vector for expression-secretion of scFv-3	this study
pLTEX-4_scFv-3	Vector for expression-secretion of scFv-3	this study
pLTEX-5_scFv-3	Vector for expression-secretion of scFv-3	this study
pLTEX-2_scFv-4	Vector for expression-secretion of scFv-4	this study
pLTEX-3_scFv-4	Vector for expression-secretion of scFv-4	this study
pLTEX-4_scFv-4	Vector for expression-secretion of scFv-4	this study
pLTEX-5_scFv-4	Vector for expression-secretion of scFv-4	this study

**Table 2:** Oligonucleotides used in this study

**Name**	**Sequence (5’ to 3’)**
(1) pre-pBR322-ori-EcoRV	CGGATATCTGAGCAAAAGGCCAGCAAAA
(2) pre-blaP-SwaI-EcoRV	CGGATATCCAATTTAAATGCGGAACCCCTATTTGTTTATT
(3) 5_EcoRV_5_ssu	TTGGATATCTTGGCGAAACGCC
(4) EcoRV_3_ssu	GATTTAGATATCGGTGAACTTTCGGG
(5) Pci_linker_sat4	GCTCTAGAGACATGTCGCCAGCGTCGACCG
(6) Pci_linker_sat5	GCTCTAGAGACATGTTGACCGACACGGCCGCTG
(7) BglII_linker_sat6	GAAGATCTGCCATGACCTCGAGGCTCGTC
(8) LEXSY_A264	CATCTATAGAGAAGTACACGTAAAAG
(9) NcoI_linker_sat6	GCTCTAGAGCCATGGTGACCGACACGGCCGCTG
(10) NcoI_linker_sat7	GCTCTAGAGCCATGGCGACCGACACGGCCGCTG
(11) LMB3	CAG GAA ACA GCT ATG AC
(12) LEXSY_scFv_KpnI	GGTGGGTACCCCGTTTGATTTCCACCTTGGTC

### Cloning of antibody fragments

Individual scFv’s were PCR-amplified from the original clones in vector pIT2 using primer 11 and 12 (adding cutting site KpnI). The PCR-fragments (729 bp) were digested with NcoI and KpnI for cloning, ligated into cut vector backbones and transformed into DH10B. Vectors pLTEX-2 and pLTEX-3 were digested with PciI and KpnI, while vectors pLTEX-4 and pLTEX-5 were digested with NcoI + KpnI. Transformants were picked from Amp^R^ agar-plates and sequence verified. Altogether, 16 different scFv-containing vector constructs were designed (Table 1).

### *E. coli* growth conditions and cell transformation

*E. coli* strain ‘ElectroMAX^Tm^ DH10B^Tm^ (Invitrogen; Cat.-No. 18290–015) was grown at 37°C with 160-180 rpm in liquid 2YT growth medium [16 g/l bacto-tryptone (Becton, Dickinson and Company), 10 g/l bacto-yeast extract (Becton, Dickinson and Company), 5 g/l NaCl (Calbiochem); resuspended in Millipore water] supplemented with ampicillin (100 μg/ml; Roth), if necessary. Electro-competent cells were transformed with 1 μl (50-200 ng/μl) purified plasmid-DNA (QIAprep Spin Miniprep Kit; Qiagen) using the ‘Micropulser Electroporator’ (BioRad) at 1.8 kV, 5 msec. For clonal selection, agar-plates were prepared by adding 15 g/L bacto-agar-agar (Becton, Dickinson and Company) to 2YT growth medium, plating cells and incubating them over night at 37°C.

### *L. tarentolae* growth conditions and stable transfection

*L. tarentolae* strain P10 (Jena Bioscience) was grown at 26°C under standard air-supply in the dark in LEXSY BHI liquid medium (Jena Bioscience) with porcine Hemin, penicillin/streptomycin (Pen/Strep) and Nourseothricin (NTC; 100 μg/ml), if necessary (all additives: Jena Bioscience). Strain P10 was passaged twice a week by inoculation of new growth medium in flat flaks (static) with a dilution of 1:30. Electroporation of cells was carried out in a prechilled 2 mm electroporation cuvette using a Gene Pulser II with pulse controller and capacitance extender plus (Biorad). For transfection, 5-10 μl purified and linearized plasmid-DNA (50-150 ng/μl) was added to 390-395 μl densely grown cells (over night as agitated suspension culture (125 rpm); OD_600_ >1.5). Cells were pulsed once at 0.45 kV and 450 μF to get 5-6 msec pulsing time with ~0.45 kV and ~20Ω, chilled shortly and cultured stationary over night at 26°C. On the next day, the majority of the culture was dispensed onto LEXSY BHI agar plates, while the rest was cultivated in flat flaks with new selection pressure (NTC) to get multi-clone cultures. After 6–10 days, single clones were picked and grown in liquid culture by continuously increasing the volume from 0.1 ml to 10 ml in LEXSY BHI growth medium. Finally, transfection success was verified for integration of the expression cassette into the host chromosome by analytical PCR as described [4].

### Production of scFv’s expressed in *Leishmania tarentolae*

Positive transfectants were used for scFv-production in 100 ml of LEXSY BHI medium containing Hemin, Pen/Strep and NTC. For this, medium was inoculated 1:30 with densely grown cultures and cultivated in agitated suspension culture (125 rpm) for 2–3 days at 26°C until OD_600_ 2.5-4.5 was reached. Cells were harvested by centrifugation for 18 min, at 1811 g and 4°C. Pellet and supernatant were separated. 10 ml/l PMSF (0.1molar; Phenylmethanesulfonyl fluoride, Sigma-Aldrich; inhibits proteases) and 1 ml Ni-NTA agarose (Invitrogen) were added to 100 ml supernatant and rotated for 2.5-3 h at 4°C. Ni-NTA was washed 2x with 5 ml washing buffer (50 mM NaH_2_PO_4_, 300 mM NaCl, 20 mM imidazole) and elution was carried out 4x with 1 ml elution buffer (50 mM NaH_2_PO_4_, 300 mM NaCl, 250 mM imidazole). All centrifugation steps were performed for 5 min, at 652 g and 4°C. Elution fractions were pooled and analysed on 4-12% Bis-Tris SDS-PAGE (Invitrogen) and western blot after electrophoretical transfer to nitrocellulose membrane. Detection of scFv’s was carried out using Protein L-HRP (Pierce) diluted 1:5000 in 2% milk powder in PBS with 0.1% Tween 20 and subsequent incubation with CN/DAB substrate (Pierce). The protein concentration was determined by gel densitometric analysis of the scFv bands in Coomassie-dyed SDS-PAGE using ImageJ (Version 1.45 s). As reference, a calibration curve on the basis of a quantifiable protein in a marker (Precision Plus Unstained Ladder, 25 kDa band, 0.12 – 1.2 μg protein) or a scFv expressed in *E. coli* was established and used for calculating the protein concentration applying linear regression equation. N-terminal scFv’s analysis by Edman degradation was undertaken by Proteome Factory (Berlin, Germany).

## Abbreviations

AA, Amino-acid; HMM, Hidden Markov Model; LEXSY BHI, Leishmania expression system brain-heart fusion medium I; MCS, Multiple-cloning site; NTC, Nourseothricin; Pen/Strep, Penicillin/Streptomycin; RE, Restriction enzyme; SAP1, Secreted acid phosphatase 1; scFv, Single-chain fragment variable; SP, Secretory signal peptide.

## Competing interests

Both authors declare that they have no competing interests.

## Authors’ contributions

SK has performed the experiments. ZK has conceived the study. SK and ZK have designed the experiments and written the manuscript. Both authors read and approved the final manuscript.
